# Prognostic microRNAs associated with phosphoserine aminotransferase 1 in gastric cancer as markers of bone metastasis

**DOI:** 10.3389/fgene.2022.959684

**Published:** 2022-08-19

**Authors:** Jingwei Ma, Meng Zhu, Xiaofeng Ye, Bo Wu, Tao Wang, Muyuan Ma, Tao Li, Ning Zhang

**Affiliations:** ^1^ The Second Department of Surgical Oncology, General Hospital of Ningxia Medical University, Ningxia, China; ^2^ College of Basic Medicine, Ningxia Medical University, Yinchuan, China; ^3^ Department of Pathology, General Hospital of Ningxia Medical University, Ningxia, China

**Keywords:** PSAT1, exosome, microRNA, gastric cancer, prognosis

## Abstract

This study analyzed PSAT1-targeted miRNAs as a prognostic predictor for gastric cancer. The relationship between the clinical manifestations of gastric cancer in patients and phosphoserine aminotransferase 1 (PSAT1) was analyzed using correlation analysis. PSAT1 was highly expressed in gastric cancer, and its low expression was associated with a poor prognosis. By pan-cancer analysis, PSAT1 could affect the tumor immune microenvironment by immune infiltration analysis. Nine microRNAs targeting PSAT1 and associated with gastric cancer were screened by miRwalk and microRNA expression in TCGA tumor tissues. Six microRNAs were obtained by survival curve analysis, including hsa-miR-1-3p, hsa-miR-139-5p, hsa-miR-145-5p, hsa-miR-195-5p, hsa-miR-218-5p, and hsa-miR-497-5p. Based on the above six microRNAs, a model for bone metastasis prediction in gastric cancer prediction was constructed. An analysis of a decision curve was performed based on the microRNAs obtained to predict bone metastasis from gastric cancer. It had a positive area under the curve (AUC) value of 0.746, and the decision curve analysis (DCA) indicated that it was clinically significant. Dual-luciferase reporter genes indicated that hsa-miR-497-5p and PSAT1 were targeted, and qRT-PCR results confirmed that hsa-miR-497-5p could down-regulate PSAT1 expression. MicroRNAs targeting the regulation of PSAT1 expression can well predict the prognosis of gastric cancer.

## Introduction

Cancer of the gastric mucosa arises from the epithelium of the mucosa, and most of its clinical manifestations are indigestion, abdominal pain, early satiety, or anorexia, but it may also present as reflux, dysphagia, and gastrointestinal bleeding (Machlowska et al., 2020). Gastric cancer is a heterogeneous and multifactorial disease caused by a combination of environmental and genetic factors, with a complex pathogenesis (Oliveira et al., 2015). Infections with *H. pylori*, nitrate- and nitrite-rich diets, smoking and drinking alcohol are all risk factors for gastric cancer (Machlowska et al., 2020; Joshi and Badgwell, 2021). Some genetic syndromes, such as CDH1, Lynch, and Peutz-Jegher syndromes, are also associated with gastric cancer (Hansford et al., 2015; Lott and Carvajal-Carmona, 2018). There were approximately 1.089 million new cases of gastric cancer worldwide in 2020, accounting for 5.6% of all cancers, making it the fifth-largest malignant tumor in the world after breast, lung, colorectal, and prostate cancers. (Sung et al., 2021). In addition, since gastric cancer exhibits insidious symptoms in the early stages, metastases are often detected at the time of diagnosis, making the prognosis poor and the mortality rate high. Globally, approximately 769,000 people will die from stomach cancer in 2020, accounting for 7.7% of all cancer-related deaths. The most common sites of metastasis for gastric cancer are the peritoneum, liver, lung, and lymph nodes, while bone metastases are rare, occurring in less than 5% of patients (Park et al., 2013; Turkoz et al., 2014). However, studies have shown that bone metastases from gastric cancer are an independent poor prognostic factor for gastric cancer and are significantly associated with overall patient survival. Bone metastases from gastric cancer have a significantly lower 5-year survival rate than non-bone metastases, with a median survival time of only about four months (Lee et al., 2007; Xiaobin et al., 2022). Because most bone metastases do not show significant clinical symptoms, the actual incidence of bone metastasis may be higher than reported. There is an urgent need for biomolecular markers that can determine the risk factors for bone metastasis in gastric cancer, assess their risk in patients, and detect early and accurately.

PSAT1 encodes the catalytic enzyme phosphoserine aminotransferase, which is associated with cell proliferation and serine anabolism (Hart et al., 2007; Montrose et al., 2021). Researchers have found that PSAT1 is aberrantly expressed in various tumor cells and promotes proliferation, metastasis, invasion, and drug resistance in a variety of malignancies, including breast, lung, and colorectal cancer (Metcalf et al., 2020; Biyik-Sit et al., 2021; Montrose et al., 2021). In addition, PSAT1 was found to promote extracellular vesicle (EV) secretion via the serine-ceramide synthesis pathway in multiple cancer types, affecting the tumor microenvironment. By activating osteoclasts, it could also promote bone metastasis. EVs are a collective term for vesicular structures encased in lipid bilayers released by various cells, including exosomes and particles. Exo is a signaling vesicle involved in normal homeostatic processes or pathological exchanges of nucleic acids, proteins, and other components between cells (Kowal et al., 2016). Exosomes not only play a role in regulating normal physiological processes, such as immune response and cell differentiation, but can also be involved in the pathophysiology of diseases, such as cancer development, progression, and metastasis (Yao et al., 2021). Tumor cells can interact with cells in the bone microenvironment through the secretion of exosomes and transfer tumor-specific contents, such as miRNA, to the bone microenvironment through exosomes, thus promoting tumor bone metastasis (Rossi et al., 2018; Tiedemann et al., 2019). Furthermore, breast cancer cells has been suggested to promote the development of breast cancer bone metastases by releasing exosomes containing miRNA-19a and IBSP (Wu et al., 2021a). However, it remains to be seen whether exosomes can regulate PSAT1.

In this study, we analyzed the clinical and tumor microenvironment of gastric cancer patients to investigate the relationship between PSAT1 and prognosis. We screened the PSAT1-targeting microRNAs with miRWalk, and then analyzed their expression in gastric cancer. Diagnosis and treatment strategies can be provided by screening marker proteins associated with gastric cancer prognosis.

## Methods

### Downloading and processing of data

We downloaded PSAT1 pan-cancer data from the UCSC Xena database for a total of 18 cancer types, including bladder uroepithelial carcinoma (BLCA), breast invasive carcinoma (BRCA), cholangiocarcinoma (CHOL), colon adenocarcinoma (COAD), esophageal carcinoma (ESCA), glioblastoma multiforme (GBM), head and neck squamous cell carcinoma (HNSC), kidney chromophobe (KICH), kidney renal clear cell carcinoma (KIRC), kidney renal papillary cell carcinoma (KIRP), liver hepatocellular carcinoma (LIHC), lung adenocarcinoma (LUAD), lung squamous carcinoma (LUSC), prostate adenocarcinoma (PRAD), rectal adenocarcinoma (READ), stomach adenocarcinoma (STAD), thyroid carcinoma (THCA), and uterine corpus endometrial carcinoma (UCEC). From the Cancer Genome Atlas (TCGA) database (https://www.cancer.gov/about-nci/organization/ccg/research), raw RNA sequencing data and clinical information were downloaded from gastric cancer patients (Wang et al., 2016; Goldman et al., 2020). Information about survival time, survival status, age, sex, tumor grade, clinical stage, pathological stage, TNM stage, OS, DSS, and PFI were collected from the patients.

### Clinicopathological and survival analysis of phosphoserine aminotransferase 1 in gastric cancer

Patients’ clinical information included age, gender, clinical stage, and TMN stage. To investigate the relationship between PSAT1 expression and clinical characteristics, we selected clinical, pathological, and TNM stages as representative outcomes with significant differences. The gastric cancer samples were then divided into high and low expression groups based on their median PSAT1 expression values. Kaplan-Meier survival curves were plotted using this method. We analyzed the relationship between PSAT1 expression and prognostic DSS (disease-specific survival) in gastric cancer taking into account the possibility of non-tumor death during follow-up. The relationship between PSAT1 expression and PFI (progression-free interval) was also examined.

### Immune infiltration analysis

CIBERSORT deconvolution algorithm is a computational method for identifying 22 types of immune cells in tissues (Bindea et al., 2013; Hänzelmann et al., 2013; Wu et al., 2021b). With R software, the CIBERSORT deconvolution algorithm was used to simulate the transcriptional features matrix of 22 immune cells, including B cells, plasma cells, T cells, natural killer cells, monocytes, macrophages, dendritic cells, mast cells, eosinophils, and neutrophils. Calculations were set at 100, and data with *p* < 0.05 were analyzed. In order to analyze the correlation between PSAT1 and immune cells, R software calculated correlation coefficients between immune cells and PSAT1. Additionally, the ESTIMATE algorithm in the R language estimation package was used to estimate the ratio of immune to stromal components in tumor microenvironments. Three types of scores were presented: immune, stromal, and ESTIMATE. Based on the correlation between PSAT1 and these three scores, we were able to analyze the correlation between PSAT1 and the tumor microenvironment. The relationships between PSAT1 expression, immune cell infiltration score, and tumor microenvironment were assessed by Spearman correlation analysis.

### Gastric cancer microRNAs associated with upregulation of phosphoserine aminotransferase 1 expression

All microRNAs that may target PSAT1 were predicted using the online miRNA target gene prediction tool miRWalk2.0 as previous researches (http://zmf.umm.uni-heidelberg.de/apps/zmf/mirwalk2) (Dweep et al., 2014; Sticht et al., 2018; Feng et al., 2021; Chen et al., 2022; Zhao and Jiang, 2022). MiRWalk integrated with several different miRNA target gene prediction tools, including miRanda, RNA22, miRDB, Targetscan, etc., which can perform multiple databases of miRNA co-screening and find the common target genes among them, maximizing the prediction confidence. Then, we conducted correlation analysis of the miRNAs that correlated negatively with PSAT1.

### MicroRNA expression and survival in gastric cancer

The microRNAs obtained above were calculated as the expression between gastric cancer tissues and normal tissues, and the microRNAs with statistically significant differences in expression were screened by the *p* < 0.05. Survival curves were plotted by Kaplan-Meier method based on the median expression value of each microRNA as previous researches (Rich et al., 2010; Lacny et al., 2015; Sun et al., 2022; Xuan et al., 2022).

### Model construction and evaluation

Using the Akaike information criterion (AIC), the optimal logistic nomogram model was constructed. We evaluated the expressiveness of the model using ROC curves and calibration curves. We also performed the Hosmer-Lemeshow goodness-of-fit test. Decision curve analysis (DCA) was used to examine the effect of the model on net clinical benefit rates at different positive thresholds. Threshold probability is the horizontal coordinate of DCA. When the nomogram model assessment value reached a certain value, bone metastasis probability was denoted as *p*.

### Cell culture and transfection

Chinese Academy of Sciences, Shanghai, provided 293T and SGC-7901 cells, which were cultured at 37 °C in a constant humidity CO_2_ incubator with DMEM + 10% FBS + 1% double antibody. Afterwards, we transfected 293T cells with 100 pmol hsa-miR-497-5p, purchased from Bioindustries, for 48 h before RNA extraction.

### RNA extraction by qRT-PCR analysis

SGC-7901 cells were treated with Trizol (Sangon, China) according to Trizol’s guidelines. In order to measure mRNA expression levels, RNA was reverse-transcribed into cDNA using Promega’s Reverse Transcription Kit (GoScriptTM Reverse Transcription Kit), followed by qRT-PCR analysis using Biotech’s qRT-PCR reagents (2X SG Fast qPCR Master Mix). As an internal reference, GAPDH was used and 2^−ΔΔCt^ was used to calculate mRNA expression levels.

### Dual luciferase reporter gene system

PmirGLO-PSAT1-3' UTR-WT and pmirGLO-PSAT1-3' UTR-MUT vector plasmids were purchased from Shanghai Sangon Biotech. 500 ng of vector plasmid and 100 pmol of hsa-miR-497-5p mimics were transfected into 293T cells and the fluorescence situation was determined 24 h after transfection using Dual Luciferase Assay Kit (Promega).

### Statistical methods

Statistical analysis was performed using the R software package (version 3.6.3). The Spearman correlation test was used to determine whether the two variables were correlated. Differences with *p* < 0.05 were considered statistically significant.

## Results

### Phosphoserine aminotransferase 1 expression and general health of gastric cancer patients

From the TCGA database, clinical information was downloaded for 375 gastric cancer patients. PSAT1 expression was divided into low and high groups based on median expression. Their median ages were 65.5 and 69, respectively, statistically significantly different (*p* < 0.05). In addition, there was a statistically significant difference in pathological types between low and high expression groups (*p* = 0.028) (Table 1 **)**. PSAT1 expression was statistically significantly different between pathological Stage IV, TNMF Stage IV, and clinical Stage IV based on patient’s clinical data [HR = 1.70 (1.09–2.68), *p* = 0.021]. There was a difference in PSAT1 expression at pathological stage IV. However, there were no significant differences in the other clinical-pathological symptoms.

**TABLE 1:** Gastric cancer patients’ demographic characteristics and PSAT1 expression.

Characteristic	Low expression of PSAT1 (*n* = 187)	High expression of PSAT1 (*n* = 188)	*p* value
Age, *n* (%)			0.032
≤65	93 (25.1%)	71 (19.1%)	
>65	93 (25.1%)	114 (30.7%)	
Gender, *n* (%)			0.073
Female	58 (15.5%)	76 (20.3%)	
Male	129 (34.4%)	112 (29.9%)	
T stage, *n* (%)			0.471
T1	10 (2.7%)	9 (2.5%)	
T2	38 (10.4%)	42 (11.4%)	
T3	90 (24.5%)	78 (21.3%)	
T4	44 (12%)	56 (15.3%)	
N stage, *n* (%)			0.638
N0	58 (16.2%)	53 (14.8%)	
N1	49 (13.7%)	48 (13.4%)	
N2	35 (9.8%)	40 (11.2%)	
N3	32 (9%)	42 (11.8%)	
M stage, *n* (%)			0.219
M0	168 (47.3%)	162 (45.6%)	
M1	9 (2.5%)	16 (4.5%)	
Pathologic stage, *n* (%)			0.112
Stage I	23 (6.5%)	30 (8.5%)	
Stage II	65 (18.5%)	46 (13.1%)	
Stage III	69 (19.6%)	81 (23%)	
Stage IV	16 (4.5%)	22 (6.2%)	
Histologic grade, *n* (%)			0.112
G1	8 (2.2%)	2 (0.5%)	
G2	64 (17.5%)	73 (19.9%)	
G3	112 (30.6%)	107 (29.2%)	
Primary therapy outcome, *n* (%)			0.769
PD	35 (11%)	30 (9.5%)	
SD	9 (2.8%)	8 (2.5%)	
PR	1 (0.3%)	3 (0.9%)	
CR	116 (36.6%)	115 (36.3%)	
Race, *n* (%)			0.062
Asian	47 (14.6%)	27 (8.4%)	
Black or African American	4 (1.2%)	7 (2.2%)	
White	118 (36.5%)	120 (37.2%)	
Histological type, *n* (%)			0.028
Diffuse Type	40 (10.7%)	23 (6.1%)	
Mucinous Type	12 (3.2%)	7 (1.9%)	
Not Otherwise Specified	103 (27.5%)	104 (27.8%)	
Papillary Type	2 (0.5%)	3 (0.8%)	
Signet Ring Type	6 (1.6%)	5 (1.3%)	
Tubular Type	24 (6.4%)	45 (12%)	
Residual tumor, *n* (%)			0.867
R0	144 (43.8%)	154 (46.8%)	
R1	8 (2.4%)	7 (2.1%)	
R2	7 (2.1%)	9 (2.7%)	

### Pan-cancer phosphoserine aminotransferase 1 expression

**FIGURE 1:**
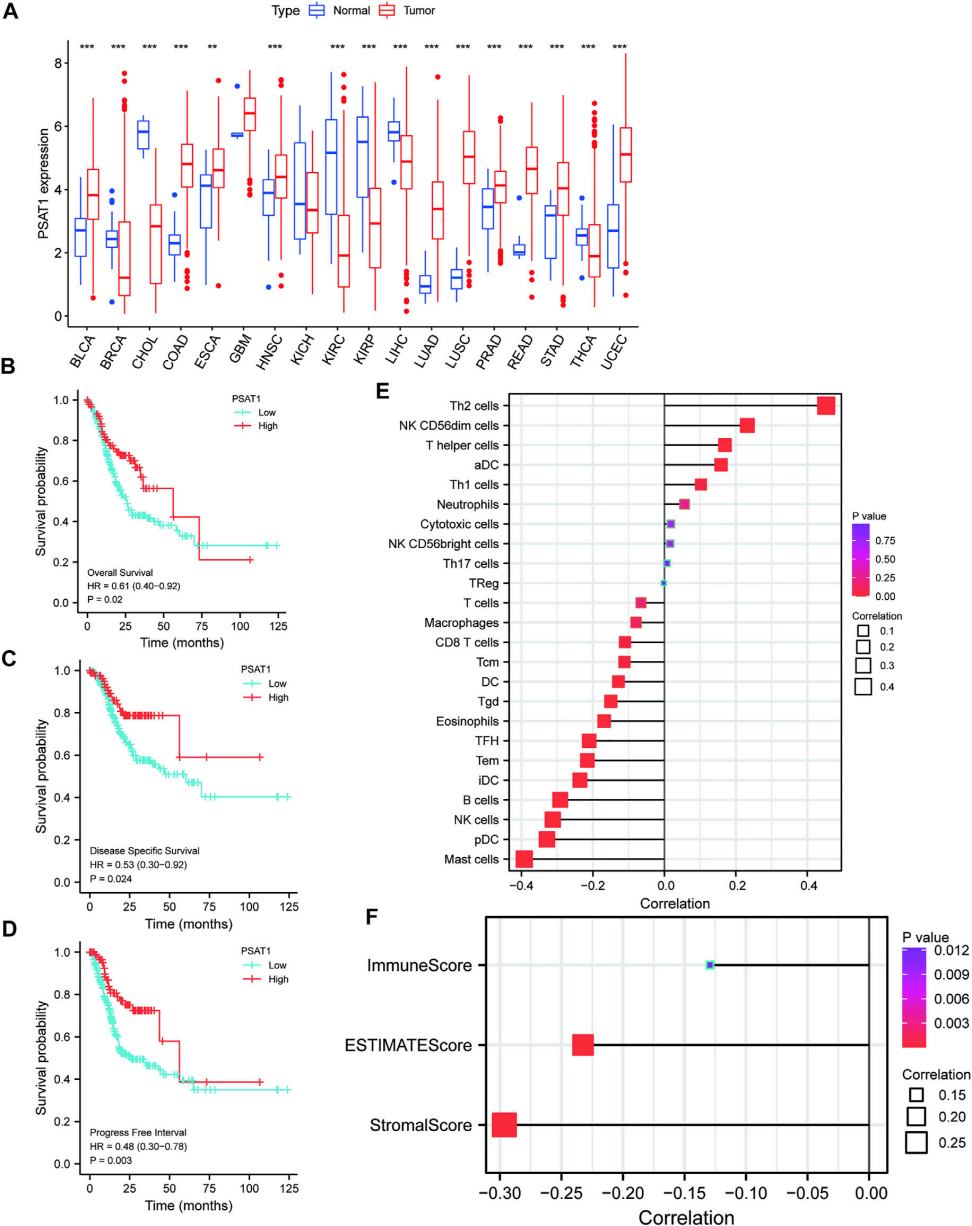
A correlation between PSAT1 expression levels and cancer patient prognosis. **(A)**. Box plot showing the level of PSAT1 mRNA expression in different cancer tissues and normal tissues; data from UCSC database; ***p < 0.001, **p < 0.01; **(B)**. Survival analysis demonstrating the overall survival of gastric cancer patients with high PSAT1 expression (OS, HR = 0.61, 95% CI: 0.40–0.92, *p* = 0.02); **(C)**. Survival analysis demonstrating the relationship between PSAT1 expression levels and DSS of gastric cancer patients (DSS, HR= 0.53, 95% CI: 0.30–0.92, *p* = 0.024); **(D)**. Survival analysis demonstrating the relationship between PSAT1 expression levels and progress-free interval of gastric cancer patients (PFI, HR = 0.48, 95% CI: 0.30–0.78, *p* = 0.003); **(E)**. Correlation analysis of PSAT1 expression with immune cell infiltration displaying that TH2 cells and NK cells are positively correlated with PSAT1, whereas plasmacytoid dendritic cells and mast cells are negatively correlated with PSAT1; **(F)**. PSAT1 correlation analysis with ImmuneScore, StromalScore, and ESTIMATEScore.

UCSC database was used to analyze PSAT1 mRNA expression levels in tumor and normal tissue samples (Figure 1A). The results disclosed that PSAT1 was highly expressed in ten cancer types relative to normal tissues, including BLCA, COAD, ESCA, HNSC, LUAD, LUSC, PRAD, READ, STAD, and UCEC. In contrast, PSAT1 expression was low in BRCA, CHOL, KIRC, KIRP, LIHC, and THCA. PSAT1 expression was not significant in GBM and KICH.

### Survival analysis of phosphoserine aminotransferase 1 in gastric cancer and immune infiltration analysis

Patients with high PSAT1 expression had significantly longer overall survival (OS, HR= 0.61, 95% CI: 0.40–0.92, *p* = 0.02), disease-specific survival (DSS, HR= 0.53, 95% CI: 0.30–0.92, *p* = 0.024), and progression-free interval (PFI, HR= 0.48, 95% CI: 0.30–0.78, *p* = 0.003) (Figures 1B–D). In gastric cancer, low expression of PSAT1 was associated with worse OS, DSS, and PFI. Therefore, patients with gastric cancer with low expression of PSAT1 had a poor prognosis.

The percentage of immune cell infiltration was calculated by Cibersort software, and samples that met the requirements were screened according to the *p* < 0.05 criterion. According to our analysis, each immune cell shows a positive correlation with PSAT1 expression, while plasmacytoid dendritic cells and mast cells have a negative correlation with PSAT1 (Figure 1E). Furthermore, ImmuneScore, StromalScore, and ESTIMATEScore were calculated using the ESTIMATE algorithm in R language estimate package. PSAT1 expression and these three scores were negatively correlated (Figure 1F). PSAT1 expression levels can influence the immune activity of tumor microenvironments, according to these results.

### Prognostic analysis of microRNAs negatively associated with phosphoserine aminotransferase 1

**FIGURE 2:**
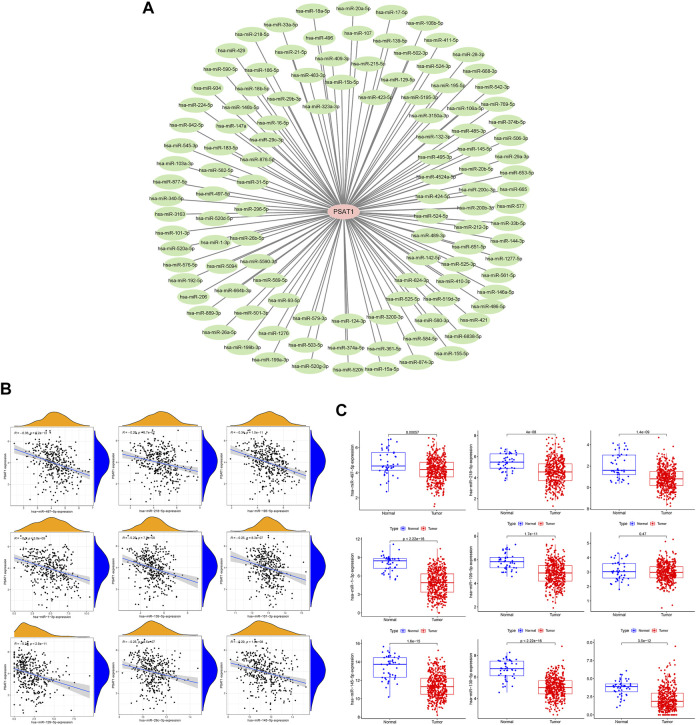
The microRNAs associated with gastric cancer expression of PSAT1. **(A)**. The miRwalk database and correlation analysis identified microRNAs that could target PSAT1 in gastric cancer; **(B)**. MicroRNAs negatively correlated with PSAT1 expression; **(C)**. Gastric tumor tissues and normal tissue samples showed negative correlations between miRNA levels and PSAT1 expression levels.

**FIGURE 3:**
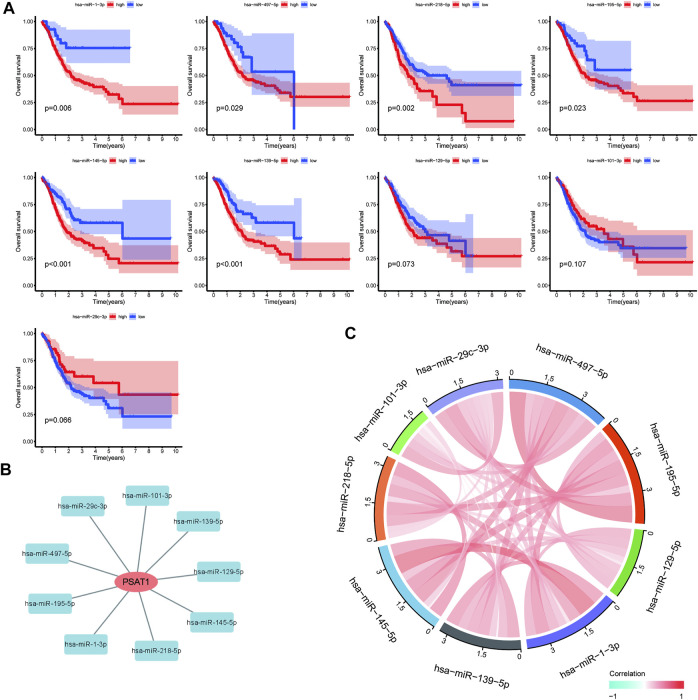
Prognostic analysis of microRNAs negatively associated with PSAT1. **(A)**. Survival analysis of miRNAs (hsa-miR-218-5p, hsa-miR-129-5p, hsa-miR-145-5p, hsa-miR-139-5p, hsa-miR-195-5p, hsa-miR-1-3p, hsa-miR-29c-3p, and hsa-miR-497-5p) significantly associated with tumor prognosis; **(B)**. PSAT1-related microRNA regulatory network; **(C)**. Correlation analysis circle diagram between PSAT1 and its negatively associated microRNAs.

MiRNAs targeting and regulating PSAT1 gene expression were identified using miRwalk database and visualized using Cytoscope (Figure 2A). We calculated the expression of the above 116 miRNAs in TCGA in gastric cancer. By analyzing the correlation between their expression and PSAT1 expression, we found that the following were negatively correlated with PSAT1 expression: hsa-miR-1-3p (r = −0.3, *p* < 0.05), hsa-miR-29c-3p (r = −0.25, *p* < 0.05), hsa-miR-101 -3p (r = −0.25, *p* < 0.05), hsa-miR-129-5p (r = −0.34, *p* < 0.05), hsa-miR-139-5p (r = −0.29, *p* < 0.05), hsa-miR-145- 5p (r = −0.29, *p* < 0.05), hsa-miR-195-5p (r = −0.34, *p* < 0.05), hsa-miR-497-5p (r = −0.36, *p* < 0.05), and hsa-miR-218-5p (r = −0.36, *p* < 0.05) (Figure 2B). Eight miRNAs were found to have low expression and be significantly different between tumors and normal tissue, namely: hsa-miR-1-3p, hsa-miR-29c-3p, hsa-miR-129-5p, hsa-miR-139-5p, hsa-miR-145-5p, hsa-miR-195-5p, hsa-miR-497-5 (Figure 2C). We calculated the *p*-value for each miRNA in relation to patient survival using KM survival curves of the eight differential miRNAs listed above. Six miRNAs were found to have significant correlations with prognosis, including hsa-miR-1-3p, hsa-miR-139-5p, hsa-miR-145-5p, hsa-miR-195-5p, hsa-miR-218-5p (Figure 3A). Then, we constructed a regulatory relationship map targeting PSAT1 using cytoscope software (Figure 3B). There was a negative correlation between these nine miRNAs (Figure 3C). Therefore, these six miRNAs (hsa-miR-1-3p, hsa-miR-139-5p, hsa-miR-145-5p, hsa-miR-195-5p, hsa-miR-218-5p, and hsa-miR-497-5p) may be associated with (hsa-miR-1-3p, hsa-miR-139-5p, hsa-miR-145-5p, hsa-miR-195-5p, hsa-miR-218-5p, and hsa-miR-497-5p) the expression of PSAT1 and the prognosis of patients.

### Construction of the nomogram predictive model and clinical utility assessment

**FIGURE 4:**
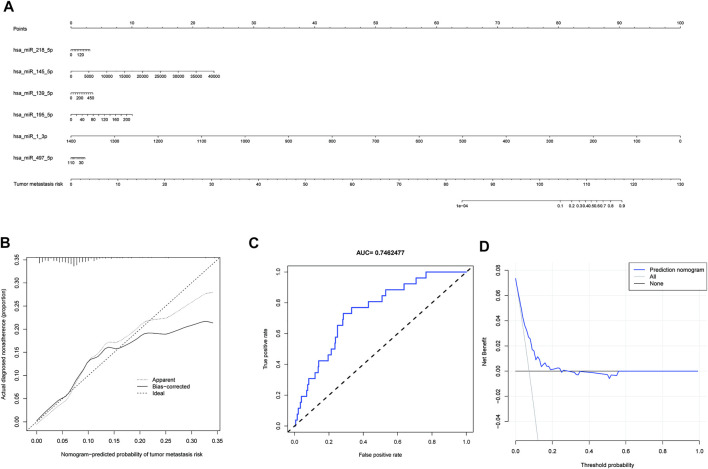
A predictive model for gastric cancer metastasis risk based on microRNAs associated with downregulated PSAT1 expression. **(A)**. Nomogram models based on six miRNAs (hsa-miR-1-3p, hsa-miR-139-5p, hsa-miR-145-5p, hsa-miR-195-5p, hsa-miR-218-5p, and hsa-miR-497-5p); **(B)**. Calibration curve for nomogram model; **(C)**. ROC curve of nomogram model (AUC = 0.746); **(D)**. Clinical decision curves illustrating the benefits of using the miRNA nomogram model.

The six miRNAs (hsa-miR-1-3p, hsa-miR-139-5p, hsa-miR-145-5p, hsa-miR-195-5p, hsa-miR-218-5p, and hsa-miR-497-5p) were built into a nomogram model (Figure 4A). Each miRNA’s corresponding scale was determined based on its actual situation in the patient. By projecting upward to the top scale points, each miRNA’s score was calculated, and the scores were summed. The risk probability of bone metastasis in the patient was calculated by projecting downward based on the total score. Validating the model, we found that it had a good AUC value (AUC = 0.746), calibration, and goodness of fit, suggesting that it could predict the risk of bone metastasis in gastric cancer (Figures 4B,C). DCA illustrating the benefits of using the miRNA nomogram model (Figure 4D). Overall, we established a predictive model for gastric cancer bone metastasis.

### Hsa-miR-497-5p targets and regulates phosphoserine aminotransferase 1

**FIGURE 5:**
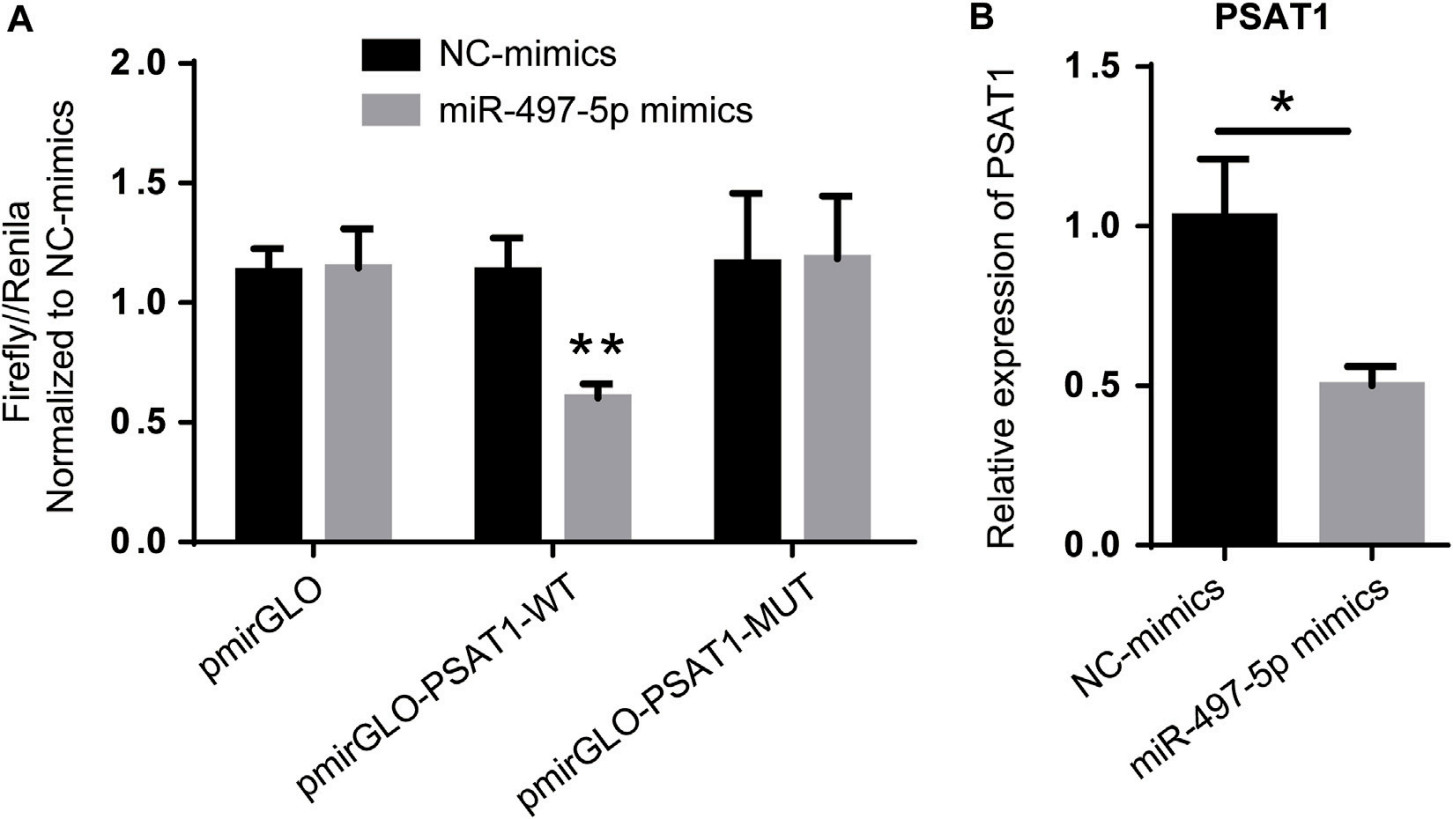
PSAT1 expression is regulated by has-miR-497-5p. **(A)**. PSAT1 and has-miR-497-5p targeting relationship revealed by dual-luciferase reporter gene system. **(B)**. The expression of PSAT1 was lower in has-miR-497-5p-treated gastric cancer cell lines compared to control cells. *p < 0.05, **p < 0.01.

Among the six miRNAs targeted by PSAT1, hsa-miR-1-3p, hsa-miR-139-5p, hsa-miR-145-5p, hsa-miR-195-5p, hsa-miR-218-5p, and hsa-miR-497-5p were most closely related to gastric cancer prognosis. PSAT1 and hsa-miR-497-5p were detected using a dual-luciferase reporter gene system and qRT-PCR. Figure 5A shows that hsa-miR-497-5p mimics reduced the fluorescence ratio compared to the control, demonstrating a targeting relationship between hsa-miR-497-5p and PSAT1. PSAT1 expression was reduced by hsa-miR-497-5p in further qRT-PCR experiments (Figure 5B). As a result of the above findings, hsa-miR-497-5p appears to be capable of targeting PSAT1 expression in order to affect gastric cancer prognosis.

## Discussion

The rapid development of precision medicine has improved the survival rate of gastric cancer by combining surgery with targeted therapy and chemotherapy, but the prognosis remains poor (Li et al., 2022). Early symptoms of gastric cancer are atypical, so early diagnosis is mainly based on endoscopic biopsy, which is a limited method. Distant metastases are often diagnosed in most patients (Dohi et al., 2017). Recently, with the continuous improvement of imaging technology, related studies have found that the percentage of patients with bone metastases from gastric cancer detected by bone scan screening can be as high as 25–45.3% (Choi et al., 1995). Several studies have demonstrated that bone metastasis, as an independent risk factor for gastric cancer, often indicated rapid deterioration of the clinical course, which seriously affected the treatment outcome and prognosis of patients (Ahn et al., 2011; Qiu et al., 2018). Moreover, patients with bone metastases may suffer from complications such as bone pain, pathological fracture, and spinal cord compression, which seriously affect their quality of life (Mikami et al., 2017). Bone metastases from gastric cancer were found to be lower than the actual rate because there were often no obvious clinical symptoms in the early stages, and skeletal screening for gastric cancer patients was not routine (Clézardin, 2017). Therefore, a predictive risk model should be developed to help detect and diagnose gastric cancer bone metastases early, enabling effective treatment plans to be developed.

PSAT1 regulates serine anabolism, playing an important role in cell proliferation, and is also essential for osteoclastogenesis (de KONING et al., 2003; Ogawa et al., 2006). Furthermore, it was found that high PSAT1 expression was closely associated with bone metastasis in malignant tumors. High expression of PSAT1 has been suggested to regulate serine anabolism, promotes osteoclast differentiation and enhances their activity, regulates the tumor microenvironment, and thus promotes bone metastasis in breast cancer (Pollari et al., 2011). PSAT1 expression was highly correlated with poor prognosis in gastric cancer in this study based on pan-cancer analysis. According to immune infiltration analysis, PSAT1 affects the tumor immune microenvironment, which indicates that PSAT1 plays a critical role in invasion and gastric cancer prognosis. Furthermore, we identified miRNAs targeting PSAT1 in TCGA tumor tissues and associated them with gastric cancer. We found that multiple miRNAs regulated PSAT1 expression (Figure 2B). The survival curve analysis identified six microRNAs, including hsa-miR-1-3p, hsa-miR-139-5p, hsa-miR-145-5p, hsa-miR-195-5p, hsa-miR-218-5p, and hsa-miR-497-5p (Figure 3A). Based on the above six microRNAs, we constructed the prognostic prediction model for gastric cancer.

MicroRNAs (miRNAs) belong to a family of non-coding RNAs of 20–24 nucleotides in length. MiRNAs are significantly associated with tumor development and metastasis (Liu et al., 2019; Wang et al., 2019; Chen et al., 2021; Zhang and Liu, 2021; Cao et al., 2022). Compared with normal tissues, miRNA expression is down-regulated in various cancers. They are widely involved in tumor metastasis and invasion by suppressing target genes and have an important role in tumor diagnosis and prognosis assessment (Daoud et al., 2019). Previous studies found that all six microRNAs used to construct predictive models were associated with malignant tumorigenesis, invasion, or metastasis. Hsa-miR-139-5p expression was low in various tumor tissues, including gastric cancer, liver cancer, and thyroid cancer (Yang et al., 2013; Montero‐Conde et al., 2020; Chi et al., 2021). Bioinformatics analysis revealed that hsa-miR-139-5p was closely associated with gastric cancer prognosis (Wang et al., 2022). Furthermore, hsa-miR-139-5p/MYB axis has been suggested to promote the proliferation, invasion, and metastasis of gastric cancer (Xie et al., 2021). The expression of hsa-miR-145-5p was down-regulated in various tumor cells, including gastric cancer, and the down-regulation of hsa-miR-145-5p expression was associated with lymph node metastasis and distant metastasis in gastric cancer, suggesting a poor prognosis (Hang et al., 2018). In addition, it was found that the exosomes secreted by ovarian cancer cells also contained hsa-miR-145-5p, and its abnormal expression was associated with distant metastasis of cancer cells (Hang et al., 2018). Hsa-miR-195-5p is also a suppressor of multiple tumor types, and its dysregulated expression is involved in the development of multiple tumors and is associated with poor tumor prognosis and drug resistance (Jin et al., 2018; Rezaei et al., 2019). It has been suggested that hsa-miR-195-5p was involved in regulating the invasion and metastasis of gastric cancer cells by binding to PD-L1 and regulating E-calmodulin expression, which was closely associated with poor prognosis for patients with gastric cancer (Zou et al., 2019; Liu et al., 2020; Liu et al., 2021). Expression of hsa-miR-218-5p is downregulated in various malignancies, including gastric, prostate, and cervical cancers, and is associated with tumor invasion and migration (Gao et al., 2009). Researchers found that hsa-miR-218-5p regulated KIT protein expression and inhibited proliferation and invasion of gastrointestinal mesenchymal tumors (Fan et al., 2014). Upregulation of hsa-miR-218-5p expression inhibits cancer progression in cervical and bladder cancer, by reducing cell migration and invasion (Chiyomaru et al., 2012; Yamamoto et al., 2013). Hsa-miR-497-5p expression is down-regulated in gastric, hepatocellular, and colorectal cancers, and is also associated with tumorigenesis, invasion, and poor prognosis (Falzone et al., 2018; Liu et al., 2021; Tian et al., 2021). In addition, abnormal expression of hsa-miR-1-3p is associated with poor prognosis of malignant tumors, such as breast cancer and small cell lung cancer (Li et al., 2020; Yan et al., 2021). Gene expression levels are closely connected to tumor metastasis according to the prediction model obtained in this study, which implicates genes involved in cancer development and metastasis. Furthermore, we developed and validated a prognostic prediction model using nine microRNAs for gastric cancer and found that AUC value with good calibration and good fit was 0.746 (Figures 4B,C). A good prediction of the risk of bone metastases in patients with gastric cancer could be obtained through this model, and it could have some application in the assessment of gastric cancer prognoses.

## Conclusion

Gastric cancers expressed high levels of PSAT1, and low levels were associated with poor prognoses. Furthermore, microRNAs targeting PSAT1 can predict gastric cancer prognosis and bone metastasis risk. Based on the results of this study, it can be concluded that the prediction model provides good predictive value in the risk assessment of bone metastasis in gastric cancer, in addition to showing some clinical application in the prognosis evaluation of gastric cancer. The study has, however, some limitations. To validate the findings of this study, a larger sample size is required. It is also necessary to conduct further experimental studies in order to clarify the specific mechanisms involved in regulation.

## Data Availability

The datasets presented in this study can be found in online repositories. The names of the repository/repositories and accession number(s) can be found in the article/supplementary material.
